# Binaural pitch fusion in cochlear implant users is broader and narrower with coherent and incoherent envelope modulation, respectively

**DOI:** 10.1121/10.0046640

**Published:** 2026-09-23

**Authors:** Yonghee Oh, Lina Reiss

**Affiliations:** 1 Department of Otolaryngology–Head and Neck Surgery and Communicative Disorders, University of Louisville, Louisville, Kentucky 40202, USA; 2 Department of Otolaryngology—Head and Neck Surgery, Oregon Health and Science University, Portland, Oregon 97239, USA

## Abstract

This study examined whether interaural amplitude modulation (AM) coherence influences binaural pitch fusion in cochlear implant (CI) users. Fourteen adults, including seven bimodal and seven bilateral CI users, completed fusion-range measurements using dichotic stimuli under unmodulated, coherent AM and two incoherent AM conditions involving interaural AM-phase and AM-rate mismatches. Coherent AM consistently broadened fusion ranges, often doubling those in the unmodulated condition, even with shallow modulation depth. In contrast, incoherent AM, including interaural phase or rate mismatches, narrowed fusion. These findings indicate that temporal-envelope coherence facilitates binaural integration in CI users, emphasizing its importance for auditory processing and CI sound-processing design.

## 1. Introduction

Binaural pitch fusion refers to the perceptual phenomenon where the brain integrates dichotically presented stimuli differing in pitch across the ears into a single and unified auditory percept. The converse scenario is fission where such stimuli are perceived as two or more distinct, non-unified percepts. Although the neural mechanisms underlying binaural pitch fusion remain unclear, the degree of binaural fusion reflects the auditory system's capacity to group or segregate different sounds. Insufficient binaural fusion prevents the integration of related inputs across the ears into a coherent perceptual experience (Aronoff *et al.*, 2025a), whereas excessive binaural fusion, the unintended grouping of unrelated inputs, is strongly predictive of speech-in-noise difficulties as it impairs the ability to segregate and understand individual talkers (Oh *et al.*, 2022). Thus, appropriate auditory grouping within specific spectrotemporal regions is essential for navigating complex acoustic environments; it allows the auditory system to “bind” various spectral components of a single sound source while effectively segregating them from competing background noise (Bregman *et al.*, 1985; Darwin, 1981).

Previous studies have shown that binaural pitch fusion depends heavily on the spectral proximity of the sounds (Aronoff *et al.*, 2025a; Oh and Reiss, 2017, 2018, 2020); however, other research suggests that temporal cues play an equally important role in this process (Aronoff *et al.*, 2025b; Oh and Reiss, 2018; Sayers and Cherry, 1957). For example, Oh and Reiss (2018) reported that the interaural coherence of temporal cues across the ears influences binaural pitch fusion in both normal-hearing and hearing aid (HA) users. Specifically, when coherent amplitude modulation (AM) is applied binaurally, the range of carrier frequencies that can be fused into a single sound (i.e., fusion range) broadens. Conversely, temporal incoherence, such as mismatches in the timing (or phase) or rate of amplitude fluctuations, suppresses fusion, which acts as a strong cue for segregation and thus narrows down the fusion range. Similarly, Aronoff *et al.* (2025b) showed that reduced interaural temporal coherence can reduce binaural fusion in normal-hearing listeners. It should be noted that in the present manuscript, we use the term temporal-envelope coherence in the auditory-grouping sense, referring to the degree to which temporal-envelope fluctuations across ears provide a common or aligned cue for grouping (Bregman, 1990; Shamma *et al.*, 2011). This usage is distinct from conventional interaural coherence in binaural hearing, which is typically quantified as the peak of the normalized cross correlation function across multiple interaural time lags. Thus, the present study does not test whether conventional coherence-based models are incorrect. Rather, it examines whether instantaneous temporal-envelope coherence, operationalized as moment-by-moment envelope alignment across ears, influences binaural pitch fusion in CI users.

Although the effects of temporal-envelope coherence have been studied in listeners with acoustic hearing, it remains unclear whether CI users' heavy reliance on temporal-envelope cues, necessitated by degraded spectral resolution, makes them uniquely sensitive to changes in modulation coherence. Thus, the current study was designed to investigate the specific impact of AM coherence on binaural pitch fusion in both bimodal CI users who use a HA in the contralateral ear, and bilateral CI users. In the current study, temporal characteristics in electric stimulation (such as phase and rate of modulation) were directly controlled *via* research interfaces, allowing isolation of the effects of temporal-envelope coherence from the spectral blurring usually introduced by clinical sound processors. This approach provides a precise mapping of how temporal-envelope mismatches affect the breadth of the “fusion range,” defined as the frequency/electrode range over which different pitches are heard as one. In addition, it is necessary to determine whether the “broad” fusion often observed in CI users is primarily broad because of peripheral factors, such as channel interaction and reduced spectral resolution, or whether it can also be modulated by temporal-envelope cues. If fusion ranges change notably with AM coherence, and in particular decrease with AM incoherence, this would suggest that broad binaural fusion is not solely a by-product of poor spectral resolution at the electrode-nerve interface. In this study, both bimodal and bilateral CI users were included because binaural pitch fusion has been shown to be abnormally broad for both groups but that the pattern and effects may differ depending on whether the two ears receive acoustic-electric or electric-electric input (Oh and Reiss, 2017, 2018, 2020). Including both groups therefore allowed us to test whether temporal-envelope coherence affects fusion across two clinically common configurations and also examine whether these effects generalize across acoustic-electric and electric-electric stimulation.

Based on previous findings in HA users (Oh and Reiss, 2018), we hypothesized that coherent AM would significantly broaden fusion ranges in both CI groups compared to unmodulated stimuli. We further predicted that even small amounts of modulation (low AM depth) would be sufficient to trigger this expansion, reflecting a high sensitivity to common temporal envelopes in CI users. This rationale is grounded in the idea that common modulation serves as a “unifying” signal that tells the brain to treat mismatched inputs as a single object. Finally, we hypothesized that interaural temporal-envelope incoherence, introduced through interaural mismatches in modulation phase or modulation rate, would act as a “segregating” cue, narrowing the fusion range. By testing these hypotheses, we aim to clarify the role of temporal-envelope coherence in supporting binaural grouping and segregation.

## 2. Methods

### 2.1 Participants

Fourteen CI users (7 bimodal CI users, ages 32 to 73, 5 females and 7 bilateral CI users, ages 30 to 66, 5 females) participated in this study. All CI users used a standard 22-electrode CI array (Cochlear Ltd., Sydney, Australia) in their CI ears. Detailed demographic data including age, gender, duration of CI uses, CI internal device, and etiology of hearing loss are shown in Table 1 in the supplementary material. In addition, individual audiograms are shown for both bimodal CI and bilateral CI listeners in Fig. S1 in the supplementary material. Unaided audiometric thresholds for non-implanted ears [dotted line in Fig. S1(A) in the supplementary material] were obtained for the bimodal CI user group, and sound field thresholds for CI ears were presented for both CI user groups. Note that the unaided thresholds for the implanted ears were not measured because of the assumption of a total loss of hearing. All HAs for bimodal CI users were verified to meet National Acoustics Laboratories (NAL)-NL2 (National Acoustics Laboratory, Australia) targets (speech stimuli at 50, 65, and 75 dB sound pressure level) using real-ear measurements to provide suitable amplification for a listener's hearing loss. All CI subjects in the study had at least one year of experience with their CI and/or HA devices and used their hearing devices on a regular basis.

**Fig. 1.**
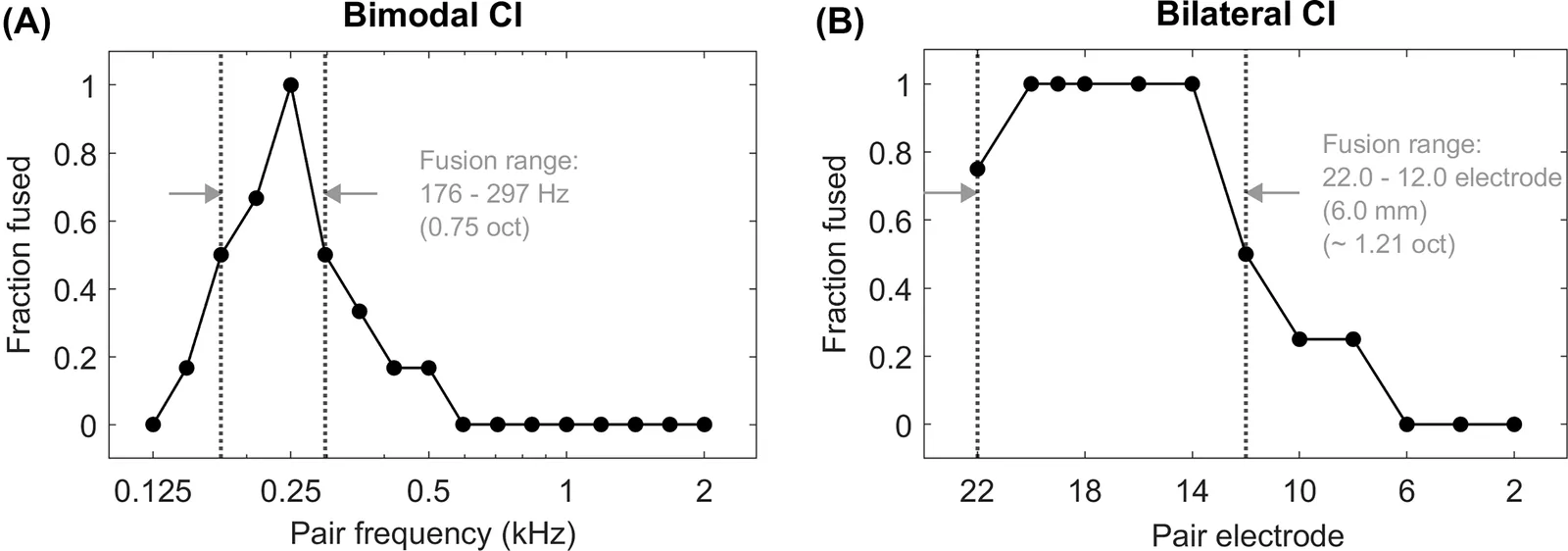
(A) Unaided audiograms for non-implanted ears (dotted lines) and sound field thresholds for implanted ears (solid lines) of the bimodal CI subjects, (B) sound field thresholds for the bilateral CI subjects, with solid and dashed lines showing individual thresholds for the left and the right ears, respectively. Note that all sound field thresholds were measured with subject's own CI processors.

All subjects were screened by monaural word intelligibility scores of 65% or higher on the Consonant Nucleus Consonant (CNC) word test with both ears/devices. They were also screened for normal cognitive function using the Mini-Mental Status Examination (MMSE) with a minimum score of 25 of 30 required to qualify (Folstein *et al.*, 1975; Souza *et al.*, 2007), ruling out cognitive impairment that would potentially influence performance. Tympanometry was also conducted to verify normal middle ear function in the non-implanted ears for the bimodal CI users. Both ethical and methodological approvals were obtained from the Institutional Review Board (IRB) of Oregon Health and Science University (OHSU). All subjects provided written informed consent.

### 2.2 Stimuli

All experiments were conducted in a double-walled, sound-attenuated booth. For CI stimulation for both CI user groups, electric stimuli were delivered *via* computer directly to the CI using Neuroelectrics Instrument Controller (NIC)2 CI research software (Cochlear Ltd, Sydney, Australia) with L34 speech processor(s) *via* the programming pod interface. Note that the current study used direct electrical stimulation through the research interface, which allows us to selectively stimulate one electrode at a time. Only even-numbered electrodes were used for the experiment to limit the number of trials. All subjects had all activated electrodes from electrode 1 to 22 and used the standard frequency-to-electrode allocations in their clinical programs.

Direct stimulation of each electrode consisted of a pulse train of 25 *μ*s biphasic pulses presented at 900 pulses per second (pps)/electrode in their everyday programs. The electrode ground was set to monopolar stimulation with both the ball and plate electrode active [monopolar simulation 1 + 2 (MP1 + 2)]. For acoustic stimulation for the bimodal CI subjects, acoustic stimuli were delivered to the non-implanted ear using an ESI Juli sound card (ESI Audiotechnik, Leonberg, Germany), TDT PA5 digital attenuator with HB7 headphone buffer (Tucker-Davis Technologies, Alachua, FL), and Sennheiser HD-25 headphones (Sennheiser Electronic, Wedemark, Germany). Each headphone's frequency response was equalized using calibration measurements obtained with a Brüel & Kjaer sound level meter (Brüel & Kjær Sound & Vibration Measurement, Nærum, Denmark) with a 1 in. microphone in an artificial ear. All acoustic stimuli consisted of sinusoidal pure tones with 10 ms raised-cosine onset/offset ramps. It should be noted that the timing cues of all stimuli were controlled by a computer and bilaterally synchronized.

To investigate how temporal-envelope coherence influences fusion, the following four distinct envelope conditions were compared. In the unmodulated (control) condition, both reference and comparison stimuli had an unmodulated envelope with constant amplitude [Fig. S2(A) in the supplementary material]. In the coherent AM conditions, the stimulus envelopes were amplitude modulated with matched AM rate across ears, whereas AM depth varied across conditions [e.g., Fig. S2(B) in the supplementary material]. The AM rate was fixed at 4 Hz, and the AM depths varied between 20% and 100% in steps of 20% points (i.e., 20%, 40%, 60%, 80%, and 100%). Here, the 4 Hz AM rate was chosen because low-frequency modulations near the syllabic rhythm of speech are important for speech perception and intelligibility (Füllgrabe *et al.*, 2009).

Two incoherent AM conditions were used. In the first incoherent AM condition [e.g., Fig. S2(C) in the supplementary material], interaural phase differences were varied between 45° and 180° in steps of 45° (i.e., 45°, 90°, 135°, and 180°). At the 4 Hz AM rate, these interaural phase shifts resulted in ongoing envelope interaural time differences (ITDs, e.g., ∼31 ms for a 45° shift) while maintaining a 0 ms onset ITD across all conditions. These delays far exceed the 5–10 ms echo threshold and the natural ITD range for localization (Blauert, 1997; Litovsky *et al.*, 1999). Thus, although these large delays were intentional to provide unambiguous grouping/segregation cues (Bregman, 1990), they do not simulate natural spatial offsets. As briefly mentioned in the Introduction, in the binaural literature, interaural coherence is traditionally defined by the peak of the cross correlation function (e.g., Bernstein and Trahiotis, 2017). Here, we use temporal-envelope coherence in a broader auditory-grouping sense to refer to the degree of moment-by-moment alignment between the envelopes delivered to the two ears, i.e., the correlation at zero lag. To avoid confusion with the conventional cross correlation–based metric, we refer specifically to conventional interaural coherence when discussing the peak cross correlation measure. Under this framework, interaural AM-phase offsets reduce instantaneous temporal-envelope coherence, even though phase-shifted periodic envelopes may remain highly coherent in the conventional cross correlation sense when temporal lag is allowed. These manipulations altered the moment-by-moment alignment of the envelopes delivered to the two ears, thereby changing the ongoing temporal cues available for binaural grouping or segregation.

In the second incoherent AM condition [e.g., Fig. 2(D) in the supplementary material], different AM rates were presented to each ear (i.e., a fixed 4 Hz AM stimulus in the reference ear and stimuli with 3, 5, or 7 Hz AM rates in the comparison ear) with randomization of the starting phases of the modulator envelopes on each trial. The modulation depth was fixed at 60% for both incoherent AM conditions. For descriptive comparison with conventional binaural metrics, normalized interaural cross correlation functions were calculated for the mean-removed AM envelopes in the AM-rate-difference conditions, and both the maximum cross correlation value and corresponding lag were extracted over the 1500 ms stimulus duration. Because the starting phases of the AM-rate mismatch stimuli were randomized across trials, cross correlation values were calculated across 1000 randomized phase pairings and then averaged within each AM-rate condition. This analysis was used to compare the behavioral fusion-range results with conventional envelope coherence/cross correlation metrics. The specific details regarding the selection and designation of the reference and comparison ears for each subject group are described in Sec. 2.3.

### 2.3 Procedure

Prior to all experiments, loudness balancing was conducted using a method of adjustment. First, the level of the electric stimulation for each electrode in the reference CI ear was initialized to a “medium loud and comfortable” current level corresponding to 6 or “most comfortable” on a visual loudness scale from 0 (no sound) to 10 (too loud). Then, for bimodal CI subjects, acoustic tones (0.125–4 kHz) were presented to the contralateral, non-implanted ear and set to medium loud and comfortable levels again using the same loudness scale as for electric levels. Tone frequencies that could not be presented loud enough to reach a medium loud and comfortable level because of too much hearing loss at those frequencies were excluded; this determined the upper limits of the loudness-balanced frequency range of the acoustic ear. For bilateral CI subjects, all contralateral CI electrodes were loudness-balanced sequentially with the reference electrodes. The electrodes/frequencies and order of presentation were randomized to minimize the effect of biases such as time-order error and underestimation or overestimation of the loudness (Marks and Florentine, 2011). This loudness balancing procedure was performed to minimize the use of loudness-difference cues and maximize focus on pitch differences as the decision criteria.

For the dichotic fusion range measurement, both reference and dichotic comparison stimuli were presented simultaneously in a 1500 ms single interval in which subjects were asked to indicate whether they heard a single fused sound or two different sounds in each ear. If subjects heard a sound only in one ear (lateralization), they were instructed to indicate that they heard one sound, as lateralization is an indication of fusion. Note that several training trials were given prior to testing to familiarize the listeners with the fusion task. For the bimodal CI subjects, the reference electrode for the CI ear, designated the reference ear, was fixed at a single electrode (electrode 18), and comparison tone frequencies presented to the contralateral ear were varied in ¼ octave steps between 0.125 and 2 kHz. For the bilateral CI subjects, the ear with worse within-ear electrode pitch discrimination was designated as the reference ear with a fixed reference electrode (data not shown), and all even-numbered electrodes on the contralateral ear were used as comparison electrodes. The worse ear was assigned to be the reference ear so that the resolution of comparison electrode testing would be maximized using the better ear instead of being limited by the worse ear. Note that the worse ear was chosen based on a global pitch (electrode) ranking score rather than a local (per electrode) measurement, as described previously in Reiss *et al.* (2018). Fusion range measurements were collected for a total of 14 conditions (1 unmodulated condition, 5 coherently modulated conditions, and 8 incoherently modulated conditions). To ensure reliability, the results for all experiments were averaged with two separate runs for each condition.

Fusion functions were computed as the averages of the responses to the multiple (six to seven) presentations of each reference and comparison stimulus pair and expressed as a function of comparison tone frequency (bimodal CI users) or electrode (bilateral CI users), which has been established to be sufficient for characterizing binaural pitch fusion in previous studies (e.g., Oh *et al.*, 2023). Responses heard as either left or right higher were assigned to a value of 0, whereas responses heard as a single sound were assigned to a value of 1. Thus, averaged response values near 0 indicate comparison stimuli that did not often perceptually fuse with the reference stimulus (were heard as two sounds), whereas values near 1 indicate comparison stimuli that were often perceptually fused with the reference stimulus (were heard as one sound). Example fusion functions for a representative subject in each subject group are shown in Fig. 1 as solid lines with circle symbols. Vertical dotted lines indicate 50% points on the fusion function, and the fusion range was defined as the range between these two lines (horizontal arrows); i.e., frequencies or electrodes were fused more than 50% of the time. Fusion range is thus a measure that indicates the breadth of fusion and can be used for comparison of fusion across participants and conditions.

For the bilateral CI users, to allow comparisons across CI electrode arrays with different inter-electrode distances, fusion ranges in units of electrodes were first converted into millimeters (measured between the centers of adjacent electrodes) using the following inter-electrode distances: variable distances between 0.4 and 0.81 mm for Cochlear CI24R/CI24RE/CI512 arrays and between 0.85 and 0.95 mm for Cochlear CI422/CI522 arrays. The results in mm were then converted into an acoustic octave unit by assuming that 5 mm is approximately 1 octave observed for both the organ of Corti and spiral ganglion for 1000 Hz and up in the human ear (Greenwood, 1990; Stakhovskaya *et al.*, 2007).

## 3. Results

**Fig. 2.**
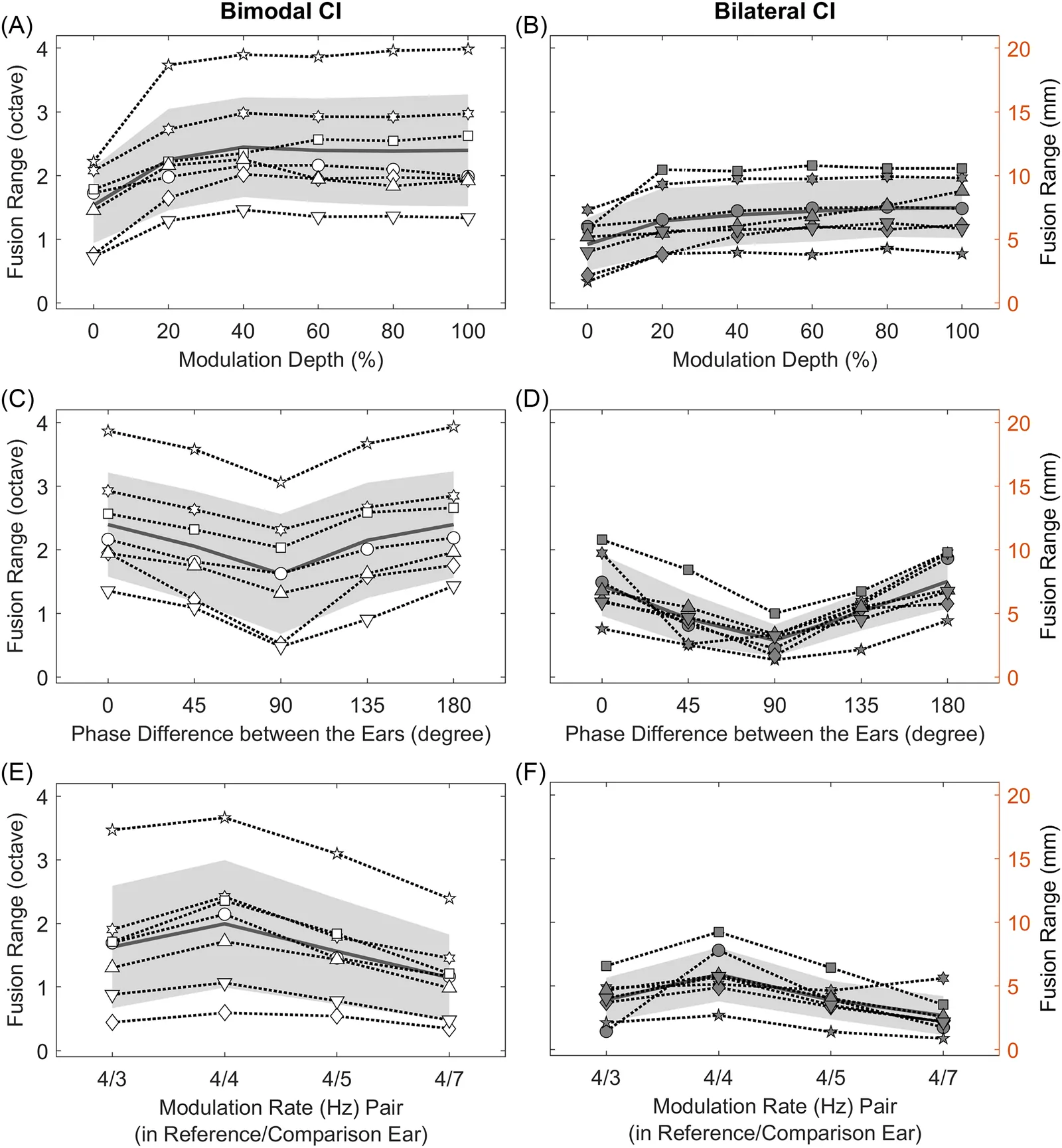
Schematic representation of the stimuli used in the four AM conditions. (A) Unmodulated condition, (B) coherent AM condition with 4 Hz AM rates in both ears, (C) interaural AM-phase difference condition with a 90° phase shift, (D) interaural AM-rate difference condition: 4 vs 7 Hz AM rate. Only stimuli with 60% modulation depths are illustrated here. Ref., reference; Comp., comparison; Diff., difference; sec, seconds.

Figure 2 illustrates the individual and mean effects of interaural AM coherence and incoherence on binaural pitch fusion in bimodal and bilateral CI users. Figs. 2(A) and 2(B) show fusion range as a function of AM depth (0%–100%), Figs. 2(C) and 2(D) show fusion ranges across interaural AM-phase offsets (0°–180° across ears), and Figs. 2(E) and 2(F) show fusion ranges across interaural AM-rate differences (reference/comparison ears: 4/3, 4/4, 4/5, and 4/7 Hz). The overall results reveal two distinct patterns: AM conditions with high temporal-envelope coherence broadened fusion ranges, whereas AM conditions with reduced temporal-envelope coherence, introduced through interaural phase or rate mismatch, narrowed fusion ranges as phase differences increased up to 90° and as AM-rate differences increased. Detailed statistical analysis results using a linear mixed model (LMM) for these conditions are provided below.

### 3.1 Effects of coherent AM on binaural pitch fusion

For bimodal CI users [Fig. 2(A)], the mean fusion range in the unmodulated condition (i.e., 0% AM depth) was 1.53 octaves. This fusion range increased with the introduction of modulation, reaching a mean of 2.44 octaves across the 20%–100% AM depth conditions but remained relatively stable across the varying modulation depths. Bilateral CI users [Fig. 2(B)] exhibited a similar upward trend: mean fusion range was 0.96 octaves at 0% depth and ranged from 1.29 to 1.49 octaves across 20%–100% depth. LMM analysis, with the fusion range as a dependent variable, AM depth (0%–100%) as a fixed factor, and the subject as a random effect, confirmed that the AM depth was a significant factor of fusion range for bimodal CI (*F*_5,30_ = 20.1, *p* < 0.001) and bilateral CI (*F*_5,30_ = 18.6, *p* < 0.001) user groups. These estimates indicate a small but reliable increase in fusion range per percentage point of AM depth and a consistent effect across subjects.

Bonferroni‐corrected pairwise comparisons indicated that fusion ranges at all nonzero AM depths were significantly larger than at the unmodulated (0%) condition in both groups (adjusted *p* < 0.001). Note that the LMM models showed good overall fit [Akaike information criterion (AIC) = 52.92 for bimodal CI; AIC = 1.13 bilateral CI]. Residual‐normality tests yielded non‐significant results (Shapiro–Wilk *W* = 0.9227 and *p* = 0.101 for bimodal CI; Shapiro–Wilk *W* = 0.9726, *p* = 0.403 for bilateral CI), providing no evidence against normality and supporting the validity of the *F*‐tests.

### 3.2 Effects of incoherent AM on binaural pitch fusion

The results showed that incoherent AM yielded non-uniform reductions in fusion ranges across both incoherent AM conditions (interaural AM-phase and AM-rate differences). First, for the AM-phase condition [Figs. 2(C) and 2(D)], the smallest mean fusion ranges occurred at the 90° interaural phase offset (1.62 octaves for bimodal CI; 0.574 octaves for bilateral CI), and the largest mean fusion ranges were observed at the 0° for bimodal CI users (2.39 octaves) and 180° for bilateral CI users (1.50 octaves). LMMs with fusion ranges as a dependent variable, AM-phase difference as a fixed factor, and a random intercept for subject, confirmed that interaural AM-phase difference significantly influenced fusion ranges (*F*_4,24_ > 21.1, *p* < 0.001 for both groups). Specifically, fusion ranges at the 90° phase mismatch were significantly narrower than those for all other phase‐difference conditions. In addition, fusion ranges at the 45° and 135° phase mismatches were significantly narrower than those at the 0° and 180° phase mismatches (Bonferroni‐adjusted *p* < 0.035 for all comparisons).

Second, for interaural AM-rate differences [Figs. 2(E) and 2(F)], fusion ranges are generally reduced when the two ears receive different AM rates compared to the matched 4 Hz condition. The largest reductions occurred at the greatest AM-rate separations (reference/comparison: 4/7 Hz), where fusion ranges dropped from 1.99 to 1.15 octaves in bimodal users and 1.18 to 0.53 octaves in bilateral users. LMMs and pairwise comparisons confirmed the significance of these rate effects (*p* < 0.001 for all cases). These models also showed high goodness-of-fit (AIC > 16.75 for all cases) and met the assumptions for normality (Shapiro–Wilk *W* > 0.943 and *p* > 0.131 for all cases). For descriptive comparison with conventional binaural metrics, normalized interaural envelope cross correlation values were calculated for the AM-rate-difference conditions. The maximum normalized cross correlation values were 1.000, 0.209, 0.210, and 0.072 for the 4/4, 4/3, 4/5, and 4/7 Hz conditions, respectively, with corresponding mean lags of 0.0, −4.7, −2.7, and −5.6 ms. Thus, AM-rate mismatches substantially reduced conventional envelope cross correlation relative to the matched 4/4 Hz condition, and the lowest value occurred for the largest rate mismatch, 4/7 Hz. This pattern broadly paralleled the behavioral reduction in fusion range, suggesting that the AM-rate effects were consistent with reduced interaural temporal-envelope similarity.

It should be noted that the peak fusion magnitude was 1.0 for all subjects and conditions, except for one condition in one subject, indicating that nearly every fusion function reached 100% fusion at one or more comparison frequencies or electrodes. Therefore, the observed broadening and narrowing of fusion ranges are unlikely to reflect simple reductions in maximum fusion probability as measured in the current study. However, because a fusion probability of 1.0 does not necessarily imply an equivalent degree of perceptual fusion across conditions, differences in fusion quality cannot be excluded. Individual fusion functions across conditions are shown in the supplementary material.

## 4. Discussion and conclusion

The results of this study demonstrate that temporal envelope cues strongly modulate binaural pitch fusion in both bimodal and bilateral CI users. Coherent temporal fluctuation consistently broadened the binaural pitch fusion range by approximately 1.5 to 2 times compared to unmodulated conditions. In contrast, interaural incoherence, whether *via* phase or rate mismatches across ears, significantly reduced these ranges. In particular, the interaural mismatches in a 90° phase or a 3 Hz rate difference (7 Hz paired with 4 Hz) resulted in the most reduced fusion ranges, indicating that the pitch fusion mechanism in CI users is highly sensitive to interaural temporal disparities.

These findings are consistent with prior observations in HA users (Oh and Reiss, 2018), suggesting that sensitivity to temporal-envelope coherence is an important cue in binaural pitch fusion processing across both acoustic and electric modes of stimulation. Regarding the effects of coherent AM, the increase in fusion ranges seen in our CI cohorts (bimodal CI: 1.53–2.44 octaves; bilateral CI: 0.96–1.49 octaves) mirrors the increase observed in acoustic listeners (HA: 0.61–1.54 octaves from Oh and Reiss, 2018), where coherent modulation promotes binaural pitch fusion. The fact that even a 20% AM depth was sufficient to significantly broaden fusion ranges suggests that the auditory system can use even subtle shared temporal-envelope cues when judging whether spectrally mismatched inputs should be grouped into a single percept. Because all conditions had synchronized onsets, the observed differences across AM-phase and AM-rate conditions are consistent with the possibility that ongoing envelope relationships influenced fusion judgments after stimulus onset. However, caution is needed in interpretation as the present design did not directly measure the time course of fusion or fission. Future studies using time-resolved behavioral or physiological measures would provide insight into how onset synchrony and ongoing envelope coherence jointly influence the dynamics of binaural fusion and fission.

In addition, the narrowing of fusion ranges under incoherent conditions suggests that binaural pitch fusion is influenced by ongoing temporal-envelope relationships in addition to peripheral spectral resolution. Although binaural fusion necessarily involves central auditory processing, peripheral factors such as broad electrode excitation patterns, channel interaction, and reduced spectral resolution may contribute to the broad fusion ranges often observed in CI users. The present results suggest that these peripheral constraints do not fully determine fusion range, because temporal-envelope manipulations systematically modulated fusion judgments. Specifically, the reduction observed at the 90° phase offset suggests that interaural envelope mismatch can provide a cue for perceptual fission. The finding that many CI users continued to perceive 180° out-of-phase envelopes as fused suggests that modulation that is covarying but in opposite directions, i.e., with an instantaneous envelope correlation of −1, may still be perceived as coherent. Alternatively, the 180° out-of-phase envelopes may elicit the equivalent of large interaural level differences, which have been shown to promote binaural fusion (Aronoff *et al.*, 2025b) and would be consistent with the increased fusion observed in that condition. Overall, these results indicate that temporal-envelope coherence can modulate binaural fusion in CI users, supporting the view that both peripheral spectral factors and central processes involved in temporal grouping contribute to binaural integration and segregation in the impaired auditory system.

Several limitations of the present study should be acknowledged to interpret the findings and guide future research. A primary limitation of this study is the relatively small sample size (*N* = 14), which may limit the generalizability of the findings while remaining typical of direct-stimulation CI studies. The participants also exhibited a wide range of etiologies and durations of CI use, which could contribute to the individual variability seen in the dotted lines of Fig. 2. For instance, the “starting” fusion range in the unmodulated condition varied considerably across subjects. This variability may be because of peripheral differences in pitch resolution reflecting differences in electrode-nerve interface quality or because of differences in auditory experience, as broad binaural fusion is associated with early or longer durations of HA experience (Reiss *et al.*, 2017, 2025). In addition, because the present study was not designed to separate the effects of peripheral spectral resolution, auditory experience, and temporal-envelope sensitivity, future studies with larger samples will be needed to determine how these listener-specific factors interact with AM coherence to shape binaural fusion.

Furthermore, this study utilized direct electrical stimulation with a single channel to provide a “clean” temporal envelope to each ear. In realistic multi-channel stimulation scenarios, there will be significant current spread and channel interaction, leading to a “smearing” of envelopes across channels within an ear that is absent in a single-channel experiment. Therefore, the robust effects of AM coherence on binaural fusion observed here, although similar to those observed in normal hearing and HA users, might be attenuated when multiple signals are applied to both ears and cross-channel envelope differences are smeared because of such channel interactions. Further research with multi-channel stimulation is needed to better understand the effects of coherent and incoherent AM on binaural fusion in CI users in a real-world multi-signal scenario. In addition, although 4 Hz captures an important low-rate component of the speech envelope, natural speech contains a broader range of modulation rates, including higher rates up to approximately 20 Hz. Future work should therefore test whether the present effects generalize to more complex, speech-like modulation patterns that include both low-rate syllabic modulations and higher-rate temporal-envelope fluctuations.

Despite these limitations, the findings from the current study offer several important implications for device programming, especially for balanced sound-processing strategies. Although prioritizing the preservation of interaural temporal-envelope coherence is essential for stable binaural pitch fusion, it is equally important that these strategies accurately represent interaural incoherence. For bimodal and bilateral CI users, “envelope-matching” may help prevent the unintended perceptual segregation of a single auditory object. However, although CI processors typically accurately track acoustic envelopes for single talkers, utilizing experimental interaural AM-phase and AM-rate mismatches allows for the isolation of central grouping mechanisms that would occur with multiple talkers. Preserving these envelope differences is necessary to facilitate the segregation of competing signals, such as target speech from masking speech. This suggests that sound-processing strategies that accurately preserve both coherent and incoherent interaural cues, while accounting for the differences between single-channel psychophysical tasks and multi-channel clinical stimulation, will be critical for balancing binaural integration with effective stream segregation. Ultimately, such strategies will provide CI users with more effective binaural performance in everyday listening situations.

Beyond sound-processing algorithms, exploring the relationship between an individual's “fusion range sensitivity” and their speech-in-noise performance could provide a vital diagnostic tool for patient evaluation. Because incoherent AM (e.g., interaural phase or rate mismatches) was shown to narrow the fusion range, this mechanism likely enables the segregation of multiple voices in complex environments. Although natural multi-talker scenarios involve realistic interaural phase differences (IPDs) that are normally fused, our findings show that artificially induced incoherence, *via* exaggerated phase and rate mismatches, can still narrow the fusion range. This suggests that incoherence, whether physiological or exaggerated, provides cues that prevent perceptual blending of competing sources. Recent evidence demonstrates that abnormally broad binaural pitch fusion negatively impacts the ability to segregate multiple voices of different pitches in complex, multi-talker environments (Oh *et al.*, 2019; Oh *et al.*, 2022; Oh *et al.*, 2023; Reiss *et al.*, 2016). Consequently, listeners with “sharply tuned” pitch fusion in the presence of incoherent cues may yield greater benefits from voice-gender differences, whereas those with broad fusion may experience “perceptual blending” of competing talkers. Utilizing these AM-based fusion metrics could help clinicians individualize device recommendations and identify patients who are most susceptible to interference in multi-talker settings.

In sum, this study demonstrates that temporal envelope cues, especially AM coherence, strongly influence binaural pitch fusion in CI users, consistent with patterns previously observed in HA users. These findings indicate the importance of temporal coherence sensitivity in central auditory processing, where coherent envelope cues facilitate the integration of matched binaural information across impaired auditory systems, and, conversely, incoherent envelope cues facilitate fission of mismatched information. Clinically, these results suggest the need to preserve interaural temporal envelope cues in CI sound-processing strategies to enable binaural grouping and segregation in complex, real-world listening environments.

## Supplementary Material

See the Supplementary Material for individual audiograms (Fig. S1), a schematic representation of the stimuli (Fig. S2), and individual fusion functions and corresponding estimated fusion ranges across all experimental conditions (Figs. S3–S16).

## Data Availability

The data that support the findings of this study are available within the article and from the corresponding author upon reasonable request.
